# Nitric Oxide and Melatonin Cross Talk on Photosynthetic Machinery

**DOI:** 10.3390/molecules30102148

**Published:** 2025-05-13

**Authors:** Moon-Sub Lee, Nusrat Jahan Methela, Gun-Ho Lee, Bong-Gyu Mun

**Affiliations:** 1 Department of Crop Science, Chungbuk National University, Cheongju 28644, Republic of Korea; 2 Department of Agriculture, Noakhali Science and Technology University, Noakhali 3814, Bangladesh; 3 Department of Environmental and Biological Chemistry, Chungbuk National University, Cheongju 28644, Republic of Korea

**Keywords:** nitric oxide, melatonin, photosynthesis, abiotic stress, crop productivity

## Abstract

Nitric oxide (NO) and melatonin (MT) significantly influence photosynthetic processes by modulating redox homeostasis, chlorophyll content, stomatal conductance, and gene expression, particularly under abiotic stress conditions. This review summarizes the intricate crosstalk between NO and melatonin, focusing on their coordinated roles in regulating photosynthetic efficiency. Evidence from various plant species indicates that the application of exogenous NO and melatonin enhances chlorophyll content, photosystem efficiency (particularly PSII), and photosynthetic performance, mitigating stress-induced damage. Molecular analysis demonstrates that both molecules influence key photosynthetic gene modulating photosystems I and II, and Calvin cycle activities. Moreover, NO and melatonin collaboratively regulate stomatal movements through ABA, Ca^2^⁺, and H_2_O_2_ signaling pathways, involving genes such as *PMRT1*, *CIPKs*, and *OST1*. Experimental data from diverse plant species under stress conditions, including drought, salinity, heavy metals, and flooding, highlight their synergistic protective effects. Exploring these mechanisms further may enable practical agricultural strategies involving combined NO and melatonin treatments to improve crop resilience and productivity under increasingly challenging environmental conditions. Future research directions should emphasize unraveling detailed molecular interactions, enabling targeted biotechnological applications in crop improvement programs for enhanced global food security.

## 1. Introduction

Photosynthesis, the most fundamental and complex physiological process in all green plants, is essential for plant growth and survival [1]. It drives biomass accumulation and energy production, directly influencing crop yield and quality in agriculturally important species such as rice, wheat, and maize. In fruit-bearing plants like tomato and grapevine, photosynthetic performance affects fruit development, sugar accumulation, and ripening. Moreover, photosynthesis is the primary mechanism of atmospheric CO_2_ fixation and global oxygen release, making it indispensable not only for plant productivity but also for sustaining life on Earth. However, the photosynthetic apparatus is frequently challenged by various abiotic stresses, including drought, salinity, extreme temperatures, and high light intensity, particularly under rapidly changing climatic conditions [2]. Under such adverse conditions, plants experience significant redox imbalances [3]. Moreover, these stressful conditions disrupt cellular redox homeostasis by impairing electron transport chains in chloroplasts and mitochondria, leading to the excessive accumulation of reactive oxygen species (ROS). The overproduction of ROS, including superoxide anion (O_2_^−^), hydrogen peroxide (H_2_O_2_), and hydroxyl radicals (•OH), results in oxidative damage to the photosynthetic machinery. This oxidative stress affects various metabolic pathways, including enzyme activities, protein structures, nucleic acid integrity, membrane stability, and cytoskeletal organization [4]. To mitigate these effects, plants use several mechanisms to maintain photosynthetic efficiency [5]. One of these mechanisms, Cyclic Electron Flow (CEF), is regulated by the NDH (NADH dehydrogenase-like complex) and PGR5/PGRL1 complexes [6,7]. Another important mechanism, Non-Photochemical Quenching (NPQ), is regulated by enzymes related to TRXs (thioredoxin) [8,9]. CEF helps balance ATP/NADPH production and protects against light stress, while NPQ dissipates excess energy to prevent photodamage [10,11,12]. Notably, nitric oxide (NO) and melatonin (MT), the signaling molecules, play an important role in preventing the reduction of photosynthetic efficiency caused by abiotic stress conditions. These molecules are regulated by several mechanisms to preserve this efficiency. NO, as a gaseous signaling molecule, affects many aspects of photosynthesis regulation [13]. It regulates stomatal conductance in plants to induce stomatal closure under drought and salinity conditions, protecting plants by minimizing water loss [14]. Furthermore, NO is involved in the biochemical regulation of photosynthetic processes, especially in the repair cycle of D1, a protein essential for Photosystem II (PSII), helping to maintain its function under high light stress and protecting it from photoinhibition [15]. Melatonin is a multifunctional signaling molecule that is critical for maintaining photosynthetic efficiency through both direct and indirect mechanisms. It also plays a critical role in non-receptor-mediated activities. Melatonin directly scavenges ROS, demonstrating its antioxidant capacity and protecting cells, tissues, and organisms from oxidative stress [16]. In addition to its antioxidant function, melatonin enhances the transcription of key genes involved in the regulation of essential photosynthetic components, ensuring stable photosynthetic efficiency under environmental stress. Among the various protective strategies used by plants, melatonin (MT) stands out as an important signaling molecule that helps plants cope with environmental stress. In addition to acting as a strong antioxidant, melatonin helps keep the cell’s redox system in balance, and promoting the repair of damaged photosynthetic proteins [17]. Many recent studies have highlighted melatonin’s key role in helping plants adjust to stress conditions, allowing them to maintain photosynthetic activity and stay physiologically stable even when the environment is unfavorable [18,19]. Moreover, Recent studies have emphasized that NO and melatonin not only individually affect the physiological processes of plants in the regulation of photosynthetic electron transport chains, stomatal behavior, and antioxidant defense, but also interact and influence each other [20,21]. Interestingly, the functions of NO and melatonin are interrelated. They influence each other’s biosynthesis and signaling pathway, forming a complex process. Melatonin activates the enzyme responsible for NO biosynthesis to promote NO production, while NO regulates gene expression, which is important for melatonin synthesis. This crosstalk helps to create an optimal internal environment for maintaining photosynthetic function in a stressful environment [19,22]. Considering the recent advances in NO and melatonin studies, the roles and mechanisms of these signaling molecules in plants have been extensively and intensively investigated. In the current review, we summarize the biosynthetic pathways and functional availability of NO and melatonin in plant systems. This review especially focuses on how they regulate important physiological processes, including how they affect photosynthesis under abiotic stress. In addition, we focus on the interactive crosstalk between NO and melatonin, highlighting their synergistic effects in protecting plants against environmental stresses through the coordinated regulation of redox balance, gene expression, and stomatal behavior. Such insights are crucial for developing biotechnological tools and agricultural practices that can optimize crop productivity, particularly in the face of climate change and increasing environmental stresses.

## 2. Redox Balance in Photosynthetic Mechanism

**Figure 1:**
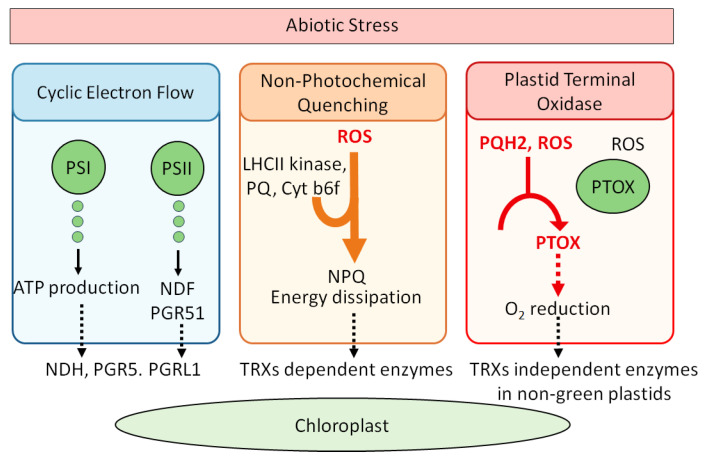
Chloroplast-based photoprotective mechanisms under stress conditions: CEF, NPQ, and PTOX pathways. Solid black arrows indicate the direction of electron flow or metabolic processes and dashed black arrows represent downstream activation or regulatory effects on specific redox-sensitive enzyme system. Orange arrow illustrates the induction of NPQ by ROS signals and red arrows show ROS-mediated activation and dashed red arrow denotes that O_2_ reduction by PTOX.

Cyclic Electron Flow (CEF) is a crucial mechanism in photosynthesis in maintaining redox balance. It redirects electrons back into the Photosynthetic Electron Transport Chain (PETC) to enhance proton pumping and boost the proton motive force (PMF) across the thylakoid membrane [23]. PMF, consisting of the proton gradient (∆pH) and the membrane potential (∆Ψ), drives ATP synthesis via ATP synthase and is subject to redox regulation [11]. Additionally, thylakoid-localized transport processes, such as the K^+^ exchange antiporter KEA3, modulate ATP synthesis and ∆pH by exporting protons from the thylakoid lumen [24]. KEA3 activity is influenced by the NADPH/NADP^+^ ratio and potentially regulated by an N-terminal cysteine residue [25]. Plants possess two distinct CEF systems—NDH and PGR5/PGRL1 complexes—that both play roles in protecting against light stress [26]. These systems contribute to balancing NADPH and ATP levels, which are critical for various biosynthetic pathways including the Calvin–Benson cycle and lipid biosynthesis. The NADPH/ATP ratio must be finely tuned to meet specific metabolic demands and integrate light energy, development, and cell catabolism. The NDH complex in *Arabidopsis* comprises 29 subunits and resembles bacterial and mitochondrial respiratory complex I [27]. It functions as a monomeric complex associated with Photosystem I (PSI) [28]. Redox regulation of NDH is complex: NTRC has been proposed to activate NDH, while TRX m4 may act as a negative regulator [29]. Although direct thiol/disulfide mechanisms for NDH regulation remain unexplored, indirect redox signaling affects NDH regulatory proteins [26]. For example, hydrogen peroxide activates NDH in barley, while low levels of ascorbic acid or reduced glutathione downregulate NDH subunit genes [30]. The role of NDH in non-photosynthetic organs, such as fruits, and its loss in some heterotrophic plants like orchids suggest a specialized function of NDH in various plant contexts [31]. The PGR5/PGRL1 complex, composed of two subunits, plays a protective role against high light stress [32]. Unlike NDH, PGR5/PGRL1 is well-characterized for redox regulation, with TRXs m4 downregulating PGR5 by reducing PGRL1 [29,33]. The complex’s regulation affects the chloroplast redox state, as evidenced by recovery in the ntrc pgr5 double mutant [34]. *Cyanobacteria* and aquatic plants like *Zostera marina* also contain CEF systems, indicating a conserved role in responding to excess radiation [7,35]. Ferredoxins (Fds) are small proteins with low redox potentials that regulate electron distribution in chloroplasts. *Arabidopsis* contains four Fd isoforms, with FD1 and FD2 implicated in CEF and Linear Electron Flow (LEF), respectively [36,37]. FDC1 and FDC2, atypical Fds with additional C-terminal extensions, may provide electrons to specific chloroplast processes, although FDC1, unlike FD1 and FD2, does not interact with FNR [18,38]. The presence of multiple Fd isoforms suggests targeted electron transfer roles. *Arabidopsis* also has multiple isoforms of PsaD and PsaE, which may serve as auxiliary docking sites for Fds, potentially influencing photosynthetic electron partitioning and redox regulation [38]. In summary, CEF systems and associated proteins like NDH, PGR5/PGRL1, and Fds are integral to balancing the NADPH/ATP ratio, managing redox states, and optimizing photosynthetic efficiency under varying conditions. Furthermore, non-Photochemical Quenching (NPQ) is crucial for preventing light-induced damage. It involves several mechanisms, including energy-dependent and zeaxanthin-dependent quenching [12,39], both of which are regulated by thioredoxins (TRXs). TRXs modulate key enzymes like violaxanthin de-epoxidase (VDE) and zeaxanthin epoxidase (ZE), influencing the interconversion between violaxanthin and zeaxanthin [8,9]. Under high light conditions, a pH gradient in the thylakoid lumen activates VDE, with TRXs providing the necessary electrons. Additionally, sustained quenching (qH) involves the lipocalin LCNP, which is negatively regulated by the TRX-like protein SOQ1 [39]. ROQH1 opposes LCNP activity to revert quenching sites back to light-harvesting states [40]. Energy distribution between Photosystems I and II (PSI and PSII) is maintained through state transitions, with the LHCII kinase (Stt7/Stn7) activated by redox changes linked to plastoquinone (PQ) and the Cyt b6f complex [41,42]. TRXs, along with other factors like CP29.3, are integral to this regulation, particularly under fluctuating light conditions [43]. Redox regulation is also crucial for the assembly of Cyt b6f and overall chloroplast function. PSII, however, is susceptible to photodamage under excess light, leading to degradation of its core protein D1. The PSII repair cycle replaces damaged D1, but photoinhibition can occur if the repair process is slower than the damage [26]. FtsH and Deg proteases are responsible for degrading D1, with FtsH activity being controlled by TRX-mediated redox changes [44,45]. In the Oxygen Evolving Complex (OEC), a disulfide bridge stabilizes the PsbO protein. TRX-mediated reduction of PsbO can destabilize it, making it more prone to degradation by Deg proteases [46]. Although the disulfide bridge is not essential for PsbO’s function, its reduction increases susceptibility to degradation, underscoring the role of redox regulation in PSII maintenance. In addition, Plastid Terminal Oxidase (PTOX) also plays a critical role beyond the PETC. It oxidizes plastoquinol (PQH2) to maintain the redox state of the PQ pool, which might generate H_2_O_2_, through reactions between superoxide anions and PQH2 [47,48]. Additionally, the role of PTOX in non-green plastids suggests that its physiological function may be linked to the absence of TRX-mediated redox regulation [49]. Overall, these components—CEF, NPQ regulation, and PTOX function—demonstrate the intricate balance plants maintain between light absorption, energy dissipation, and redox regulation to optimize photosynthetic efficiency and protect against stress. Figure 1 illustrates chloroplast associated photoprotective mechanisms under stress conditions including CEF, NPQ, and PTOX pathways.

## 3. The Relationship Between Nitric Oxide and Photosynthetic Mechanism

Nitric oxide exerts its effects on photosynthesis through multiple pathways, influencing both the biochemical and physiological aspects of the process. One of the primary ways NO affects photosynthesis is through the regulation of stomatal conductance. NO has been shown to induce stomatal closure, particularly under drought and high salinity conditions [50,51,52], by activating signaling cascades involving secondary messengers such as cyclic GMP (cGMP) and cyclic ADP-ribose (cADPR) [53,54,55]. This stomatal closure, while beneficial for reducing water loss, can limit CO_2_ uptake and supply to the mesophyll cells, thereby transiently reducing photosynthetic rates [51,56]. The dual role of NO in stomatal regulation highlights its function as a mediator that balances water conservation with the need for carbon fixation, reflecting its broader role in plant stress physiology. At the biochemical level, NO interacts with components of the photosynthetic electron transport chain (ETC) [57]. NO can modulate the activity of PSII by influencing the repair cycle of the D1 protein, which is essential for maintaining PSII functionality under high light conditions [58,59]. By preventing photoinhibition through non-photochemical quenching mechanism, NO helps sustain the efficiency of the light reactions of photosynthesis. NO has also been shown to regulate the activity of RuBisCO (ribulose-1,5-bisphosphate carboxylase/oxygenase), the enzyme responsible for carbon fixation in the Calvin cycle, primarily through post-translational modifications such as S-nitrosylation of key cysteine residues. This modification alters the enzyme’s conformation, affecting its carboxylase/oxygenase activity and thereby modulating CO_2_ fixation efficiency under stress conditions [60,61]. This regulation can have profound effects on the overall efficiency of carbon assimilation [62,63]. Furthermore, NO plays a critical role in the transcriptional regulation of photosynthetic genes. By modulating the expression of genes encoding key enzymes and proteins like glutathione reductase (GR), psbA, and psbB, NO and melatonin influence antioxidant defense and photosystem II stability. This regulation occurs through their interaction with redox-sensitive transcription factors and signaling cascades, such as MAPK and calcium-dependent protein kinases, which mediate stress-induced gene expression in chloroplasts [13,64], NO ensures that the photosynthetic machinery is optimized for both light capture and carbon fixation, particularly under fluctuating environmental conditions [58,59]. Under stress conditions, such as drought, salinity, or high temperatures, NO production is often upregulated as part of the plant’s adaptive response [65,66,67]. This increase in NO can have dual effects: while it may initially reduce photosynthetic activity by inducing stomatal closure, it also activates protective mechanisms that mitigate oxidative damage and preserve the integrity of the photosynthetic apparatus [68,69]. Thus, NO serves as both a regulator of photosynthesis and a mediator of stress responses, balancing the need for immediate survival with long-term growth and productivity.

## 4. Melatonin and Its Protective Effect on Photosynthetic Machinery

**Figure 2:**
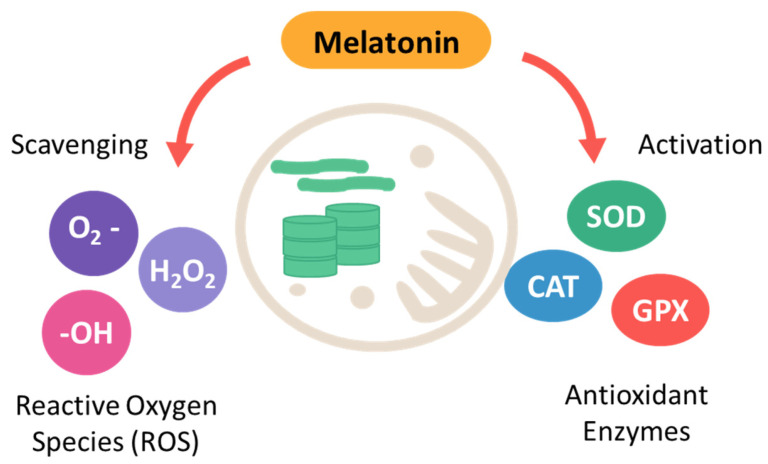
Role of melatonin in alleviating oxidative stress.

Alike NO, melatonin plays a crucial role in regulating photosynthesis, particularly under abiotic stress [70,71]. Its protective effects on the photosynthetic machinery are multifaceted, involving both direct and indirect mechanisms. A key function of melatonin is its ability to mitigate oxidative stress, which poses a significant threat to photosynthetic efficiency, especially under high light conditions [72]. Melatonin directly scavenges reactive oxygen species (ROS), safeguarding critical components like chlorophyll, thylakoid membranes, and photosystem proteins from oxidative damage [72]. This antioxidant activity is particularly important under high light conditions, where the production of ROS can exceed the capacity of the plant’s endogenous antioxidant defenses. It has been shown to upregulate the expression of genes encoding antioxidant enzymes such as SOD, CAT, and APX, as well as to increase the levels of non-enzymatic antioxidants like glutathione (Figure 2) [73,74,75].

This upregulation further strengthens the plant’s ability to cope with oxidative stress and protects the photosynthetic machinery from damage. By reducing oxidative damage, melatonin helps maintain the functionality of both photosystems and the overall efficiency of the light-dependent reactions of photosynthesis. Melatonin also influences the repair and stabilization of photosystem II, particularly under stress conditions [70,76]. For example, melatonin has been shown to accelerate the repair of the D1, D2, Lhcb1, Lhcb2, and CP43 protein, which is prone to damage under high light intensity in PSII [76,77] and PSI proteins, including Lhca1, Lhca2, and Lhca3 [71]. Current research suggests that melatonin achieves this by enhancing the transcription of photosynthetic genes like *PsbA*, *PsbB*, *PsbC*, *PsbD*, and *PsbO*, which encode essential PSII proteins [78]. By facilitating the rapid turnover of damaged D1 proteins, melatonin sustains PSII activity and prevents photoinhibition, thereby maintaining photosynthetic efficiency even under stressful conditions. Stomata, surrounded by guard cells, control gas exchange and are vital for photosynthesis and transpiration. Quantum dot nanoparticles was used to show that melatonin is present in guard cells [79]. Research indicates melatonin increases stomatal aperture size and improves traits like number, length, and width [75,80,81,82]. This effect is likely mediated by the CAND2/PMTR1 receptor, as pmtr1 mutants fail to close stomata properly, a defect corrected by maize PMTR1 in *Arabidopsis* [83,84]. ABA regulates stomatal closure by activating guard cell channels, reducing turgor, and promoting H_2_O_2_ production [85]. Li et al. (2015) [86] found melatonin lowers ABA levels by downregulating *MdNCED3* and upregulating *MdCYP707A1* and *MdCYP707A2*. Melatonin also enhances *WRKY17* expression under drought stress, suggesting it influences ABA signaling through *WRKY17* [87]. The mechanisms of melatonin’s effect on stomatal movement are not fully understood. Melatonin regulates CAND2/PMTR1-dependent stomatal closure by modulating H_2_O_2_ and Ca^2+^ signals, indicating a coordinated interaction between melatonin and ROS signaling [83,86,88]. Taken together, Melatonin may regulate stomatal movement by modulating ABA signaling or directly interacting through CAND2/PMTR1, with ROS being a key downstream factor. Recent studies suggest melatonin scavenges ROS and may also regulate critical plant processes [83]. Understanding melatonin’s role in chloroplast activities will likely require further investigation into its interaction with ROS, with CAND2/PMTR1 offering new insights into this signaling pathway. It also helps to optimize stomatal opening, ensuring sufficient CO_2_ uptake for photosynthesis while minimizing water loss. This regulation is particularly important under drought conditions, where water conservation is critical [89]. Furthermore, melatonin affects the expression of genes involved in photosynthesis and stress responses. For instance, melatonin has been shown to upregulate the expression of genes encoding RuBisCO and other enzymes involved in the Calvin cycle, thereby enhancing carbon fixation and overall photosynthetic capacity [90]. Additionally, melatonin may affect the post-transcriptional and post-translational modifications of chloroplast proteins, increasing the levels of PSI and PSII proteins and enhancing the activity of chloroplast enzymes like CLH, PPH, and Rubisco [72].

## 5. Nitric Oxide–Melatonin Crosstalk in Plant Photosynthesis and Stress Adaptation

The dynamic interplay between NO and MT in plants represents a finely tuned regulatory network essential for optimizing photosynthesis, particularly under stress conditions. This crosstalk involves a complex integration of signaling pathways that work together to maintain cellular homeostasis, ensuring plant survival and productivity. At the heart of this interaction lies the ability of melatonin to enhance NO biosynthesis. Melatonin achieves this by upregulating the expression of genes responsible for NO production and by activating key enzymes like nitric oxide synthase (NOS) and nitrate reductase (NR) [91,92]. This boost in NO production is crucial, as NO plays a vital role in various plant processes, including the regulation of stomatal conductance, which directly affects photosynthetic efficiency. Moreover, recent studies have demonstrated that nitric oxide and melatonin synergistically contribute to maintaining chlorophyll content and improving photosynthetic performance under stress conditions (Table 1). However, the relationship between NO and melatonin is reciprocal. NO also influences melatonin biosynthesis and its signaling pathways. Research has shown that NO can modulate the expression of genes involved in melatonin synthesis by inducing the expression of key enzymes, such as tryptophan decarboxylase (TDC), tryptamine 5-hydroxylase (T5H), serotonin N-acetyltransferase (SNAT), and caffeic acid O-methyltransferase (COMT), through a cyclic guanosine monophosphate (cGMP) signaling pathway [93]. This creates a feedback loop that allows plants to finely adjust their internal levels of both molecules. This feedback mechanism is essential for maintaining the balance between NO and melatonin, particularly under environmental stresses that could otherwise lead to oxidative damage in the photosynthetic machinery. One of the key areas where this crosstalk is evident is in the modulation of ROS levels. As mentioned earlier, ROS mainly produce in chloroplast, mitochondria and peroxisome [94] and melatonin is known for its potent antioxidant properties, directly scavenging ROS and preventing oxidative stress along with protecting proteins involved in chlorophyll and photosynthesis [95]. By regulating NO biosynthesis, melatonin helps to prevent the excessive accumulation of peroxynitrite, a damaging RNS that can form when NO levels are too high [16,96]. This protection is critical for safeguarding the photosynthetic apparatus from oxidative damage, ensuring that plants can continue to photosynthesize efficiently even under adverse conditions [97].

**Figure 3:**
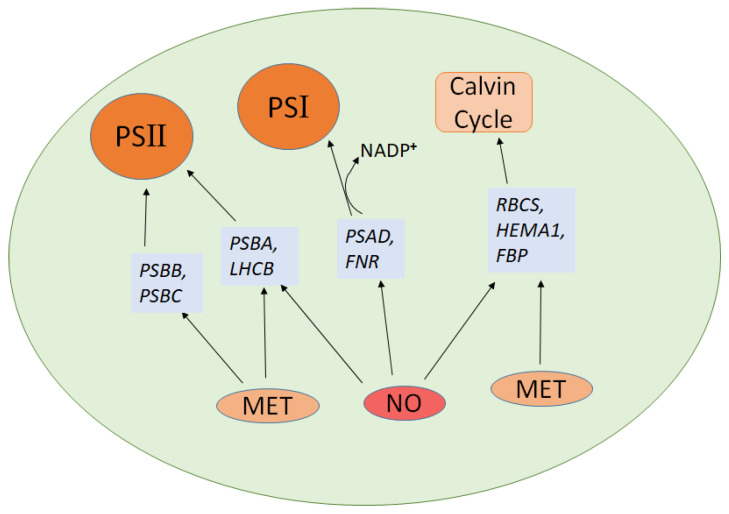
NO and melatonin associated genes in photosynthesis. The diagram illustrates how nitric oxide (NO) and melatonin (MET) influence the expression of photosynthesis-related genes. Solid black arrows indicate regulatory or activation relationships between NO/MET and specific genes involved in photosynthetic complexes and Calvin cycle function. The arrow from NADP^+^ to PSI represents electron transfer leading to NADPH formation during photosynthetic light reactions.

Nitric oxide and melatonin both play critical roles in regulating photosynthesis by modulating the expression of key genes involved in the photosystems, the Calvin cycle, and antioxidant defense mechanisms. Notably, both compounds upregulate *HEMA1*, a gene essential for chlorophyll biosynthesis, which enhances the light-absorbing capacity of plants [110,111,112]. In terms of photosystem function, both NO and melatonin upregulate *PSBA*, encoding the D1 protein of Photosystem II, and *LHCB*, responsible for light harvesting. *PSAD* plays a key role in ferredoxin-mediated NADP^+^ photoreduction on the reducing side of PSI [113]. NO upregulates *FNR* (ferredoxin-NADP^+^ reductase), enhancing NADP^+^ reduction and improving the overall efficiency of the electron transport chain [114]. MT and NO together enhanced photoprotection by activating the D1 repair pathway in PSII and improving NADP^+^ photoreduction in PSI during cold stress and NO is essential for MT-induced CO_2_ assimilation and photoprotection in cucumber seedlings [20]. These actions improve light capture and energy transfer within the photosystems. Melatonin uniquely upregulates additional photosystem genes, such as *PSBB* and *PSBC* [72], which are vital for the stability and function of Photosystem II. For the Calvin cycle, both NO and melatonin increase the expression of *RBCS* (the small subunit of Rubisco) and *FBP* (fructose-1,6-bisphosphatase), enhancing CO_2_ fixation and improving carbohydrate production [115,116]. It was reported that applying exogenous melatonin helped protect *Rhododendron*, an ornamental woody plant, from prolonged heat stress [117]. This protection was achieved by enhancing melatonin levels, boosting the electron transport rate, improving the activities of photosystem II and I, increasing rubisco activity, and elevating ATP content. Additionally, transcriptomic analysis revealed several heat-induced genes with altered expression linked to photosynthetic processes [117]. Figure 3 depicts genes related to NO and melatonin that are involved in the photosynthesis process.

**Figure 4:**
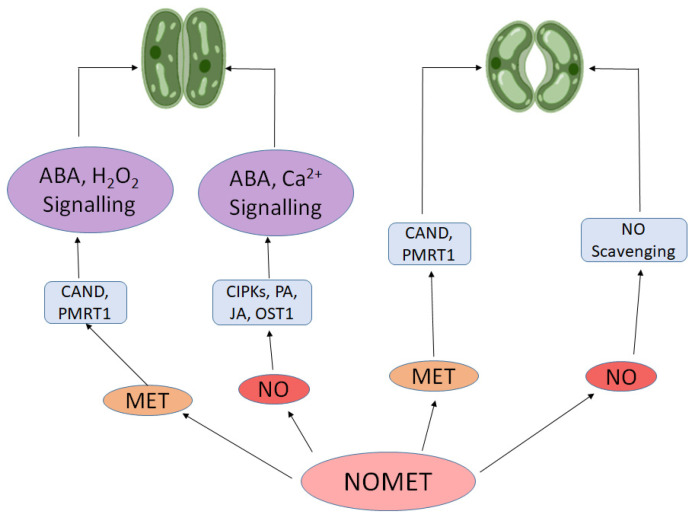
NO and melatonin induced stomatal movement. This diagram illustrates the interaction of nitric oxide (NO) and melatonin (MET) in regulating stomatal movement through hormonal signaling pathways. Solid black arrows indicate activation or signaling pathways involving abscisic acid (ABA), hydrogen peroxide (H_2_O_2_), calcium (Ca^2+^), and their downstream components. Bidirectional arrows between NO and MET represent their reciprocal regulatory effects, while the arrow from NO to “NO Scavenging” denotes its self-regulation through detoxification mechanisms.

It is important to note that the physiological effects of exogenous NO and melatonin are strongly influenced by their concentration and exposure duration. Studies have shown that low to moderate concentrations of melatonin (e.g., 50–200 µM) or NO donors such as SNP (50–100 µM) typically enhance stress tolerance and photosynthetic activity, whereas higher concentrations may induce oxidative or nitrosative stress, disrupt redox signaling, or inhibit growth [108,118]. The timing and frequency of application also play a crucial role; prolonged exposure may desensitize signal transduction pathways or interfere with endogenous hormone balance. Therefore, dose–response optimization is essential when considering the exogenous use of NO and melatonin in plant systems. Therefore, proper optimization of dose and timing is critical when considering the application of these molecules in research or field conditions. Furthermore, both NO and melatonin boost the expression of SOD (superoxide dismutase), a key antioxidant enzyme that decompose O_2_^•−^ and CAT, APX and GPX that breakdown H_2_O_2_ [119,120,121]. This action protects the photosynthetic machinery from oxidative stress, particularly under adverse environmental conditions, ensuring sustained photosynthetic performance. The all-coordinated gene expression ensures that the photosynthetic machinery remains robust and responsive to changing environmental conditions, thereby optimizing plant’s ability to adapt and thrive. Both NO and melatonin play dual role in stomatal movement. NO’s role extends beyond just influencing melatonin levels. It also plays a significant part in regulating stomatal conductance—critical for balancing water conservation with CO_2_ uptake. Under stress conditions, NO typically induces stomatal closure to reduce water loss through ABA signaling like ABA receptor, CIPKs (calcineurin B-like protein interacting protein kinase), OST1 (open stomata 1) and transcription factors [122,123]. NO also plays role in stomatal closure by small signaling peptides (CLAVATA3/embryo surrounding region (CLEs) [124], polyamines [125] and jasmonic acid [126]. Yet, melatonin can counteract this effect by re-opening stomata, thus facilitating continued CO_2_ uptake necessary for photosynthesis [86,127]. This delicate balance between NO-induced stomatal closure and melatonin-promoted stomatal opening illustrates the complementary roles these molecules play in maintaining photosynthetic efficiency. Furthermore, in *Arabidopsis*, the melatonin receptor CAND2 and PMTR1 regulates melatonin-induced stomatal closure via mediating Ca^2+^ and H_2_O_2_ signaling [88,98]. Figure 4 provides simple illustration of stomatal movement induced by NO and melatonin.

Recent research highlights the formation of N-Nitrosomelatonin (NOMET) from the interaction of melatonin with NO. Acting as a key metabolic signal, NOMET travels from roots to cotyledons in seedlings, significantly reducing oxidative and nitrosative stress [128]. Research confirms that NOMET as a better NO donor than GSNO and CySNO [22,128]. This underscores the collaborative role of NO and melatonin in enhancing plant resilience. The interplay between NO and melatonin is central to plant adaptation, with each regulating the other’s synthesis and activity to safeguard photosynthetic machinery under stress. As research advances, the synergy between these molecules is increasingly recognized as essential for plant resilience and productivity.

## 6. Conclusions and Future Perspectives

NO and MT are essential regulators of photosynthetic machinery, particularly under abiotic stress conditions. Their ability to modulate redox homeostasis, stomatal conductance, and gene expression plays a critical role in maintaining photosynthetic efficiency and ensuring plant survival under environmental challenges [129,130]. The crosstalk between NO and melatonin represents a sophisticated regulatory network that integrates multiple signaling pathways to optimize photosynthesis. Photosynthesis is the primary driver of plant growth and crop yield. Thus, factors that influence photosynthetic efficiency have significant implications for agricultural productivity. Emerging studies have demonstrated that NO and melatonin enhance plant resilience to stresses such as drought, salinity, and heat by protecting photosynthetic components from oxidative damage, preserving carbon fixation, and maintaining water-use efficiency [131]. By modulating redox balance, both molecules prevent excessive ROS accumulation, thereby sustaining photosynthetic integrity and improving crop performance under adverse conditions. Recent findings suggest that exogenous application of NO donors or melatonin, as well as genetic manipulation of their biosynthetic pathways, can lead to improved stress tolerance and yield in crops [132,133]. These strategies offer promising avenues for agricultural innovation, especially in the context of climate change and the urgent need to enhance global food security. Future research should focus on elucidating the molecular mechanisms underlying NO and melatonin signaling, particularly their interactive networks with other hormonal and redox systems. Additionally, exploring the combined application of NO and melatonin could uncover synergistic effects that further enhance photosynthetic machinery resilience beyond what either molecule can achieve alone [134]. Integrating these findings into crop breeding programs and sustainable agricultural practices could transform how crops are developed and managed to ensure food security in the face of a changing and unpredictable climate.

## Figures and Tables

**Table 1:** Nitric oxide and melatonin in chlorophyll content and photosynthesis.

Sl. No.	Plant Species	Source	Plant Physiological Process	Chlorophyll Content	Photosynthesis	Ref.
01	*Arabidopsis*	MT, SNP			DAF-FM DA and Stomatal aperture confirmed that (PMTR1) Melatonin-mediated stomatal closure is associated with increased NO levels	[98]
02	Spinach	NO and MT	Flooding stress	Chl a, b, Car	Photosynthesis efficiency (PSII), stomatal conductance (gs), chlorophyll fluroscence (Fv/Fm) increased	[99]
03	Tomato	MT and endogenous NO	Aluminium stress	Chl a, b, Car reduced		[100]
04	Tomato	MT and endogenous NO	NaCl stress	Chl a, b, Car reduced	Fv/Fm	[101]
05	Wheat	MT and endogenous NO	Cadmium stress	Total Chl reduced	Fv/Fm	[102]
06	Cucumber	MT, and endogenous NO	Salt stress		DAF-FM DA Fv/Fm net photosynthetic rate (P_n_), stomatal conductance (G_s_), intercellular CO_2_ concentration (C_i_), and transpiration rate (E_t_) reduced	[103]
07	Soybean	MT, SNP	Drought	Chl a, b		[104]
08	*Catharanthus roseus*	SNP and MT	Cadmium stress	Total Chl		[105]
09	Soybean	SNP, MT	Metal stress (Lead and cadmium)	Total Chl		[106]
10	Tomato	SNP, MT	Sodic alkaline stress	Total Chl	Pn	[107]
11	Cucumber	SNP, MT	Nitrate stress	Chl a, b, Car	PSII, Electron transport rate (ETR) and Non-photochemical quenching (NPQ)	[108]
12	Maize	MT induced endogenous nitric oxide	Lead toxicity	Total Chl	Fv/Fm	[109]

## Data Availability

No new data were created or analyzed in this study. Data sharing is not applicable to this article.
